# Acute fat loss does not affect bone mass

**DOI:** 10.1038/s41598-021-93450-y

**Published:** 2021-07-08

**Authors:** Marie K. Lagerquist, Karin L. Gustafsson, Petra Henning, Helen Farman, Jianyao Wu, Klara Sjögren, Antti Koskela, Juha Tuukkanen, Claes Ohlsson, Ingrid Wernstedt Asterholm, Louise Grahnemo

**Affiliations:** 1 Centre for Bone and Arthritis Research, Department of Internal Medicine and Clinical Nutrition, Institute of Medicine, The Sahlgrenska Academy, University of Gothenburg, Su Sahlgrenska, Vita Stråket 11, 413 45 Gothenburg, Sweden; 2 Department of Anatomy and Cell Biology, Cancer and Translational Medicine Research Unit, University of Oulu, Oulu, Finland; 3 Unit of Metabolic Physiology, Department of Physiology, Institute of Neuroscience and Physiology, The Sahlgrenska Academy, University of Gothenburg, Gothenburg, Sweden

**Keywords:** Bone, Metabolic syndrome, Experimental models of disease

## Abstract

Obesity has previously been thought to protect bone since high body weight and body mass index are associated with high bone mass. However, some more recent studies suggest that increased adiposity negatively impacts bone mass. Here, we aimed to test whether acute loss of adipose tissue, via adipocyte apoptosis, alters bone mass in age-related obese mice. Adipocyte apoptosis was induced in obese male FAT-ATTAC mice through AP20187 dimerizer-mediated activation of caspase 8 selectively in adipocytes. In a short-term experiment, dimerizer was administered to 5.5 month-old mice that were terminated 2 weeks later. At termination, the total fat mass weighed 58% less in dimerizer-treated mice compared with vehicle-treated controls, but bone mass did not differ. To allow for the detection of long-term effects, we used 9-month-old mice that were terminated six weeks after dimerizer administration. In this experiment, the total fat mass weighed less (− 68%) in the dimerizer-treated mice than in the controls, yet neither bone mass nor biomechanical properties differed between groups. Our findings show that adipose tissue loss, despite the reduced mechanical loading, does not affect bone in age-related obese mice. Future studies are needed to test whether adipose tissue loss is beneficial during more severe obesity.

## Introduction

Low bone quality and low bone mass increase the risk for fractures^1^. To resist fractures, mechanical loading of the skeleton is important as it maintains skeletal health by increasing bone mass^2^. Decreased mechanical loading, for example during bed-rest or decreased gravitational forces of spaceflight, results in bone loss^3–5^, while increased mechanical loading, induced by for example exercise, results in bone gain^2^. Mechanical loading also increases with the increasing body weight observed during for example obesity, and obesity has for long been thought to protect bone, as obesity has been associated with increased bone mass^6–8^ and decreased fracture risk^9–11^. However, obesity has also been associated with increased fracture risk^9,12–14^, indicating that the relationship between obesity and bone is complex.

Part of the complexity may be explained by the fact that the studies have used different measures of obesity. Body mass index (body weight in kg/height in cm^2^) is commonly used as a measure. However, the fact that body weight is made up of several tissues, largely muscle and adipose tissue mass, further contributes to the complexity. Muscle contributes to mechanical loading by its weight and by exerting forces on the bone through attachment to the bone via tendons^2^. As expected, association studies have shown that muscle mass is a strong positive determinant of bone mass and that low muscle mass is associated with increased fracture risk^15,16^. Adipose tissue may not only protect against fractures through increased mechanical loading, but also by providing extra padding in the event of fall^2^. However, adipose tissue is not an inert tissue; it secretes cytokines with systemic effects. During obesity, the secretion of inflammatory cytokines increases, which can lead to systemic chronic low-grade inflammation^17–20^. As inflammation is generally detrimental for bone, as observed during for example rheumatoid arthritis^21^, the obesity-associated inflammation may explain how obesity could negatively affect bone. Visceral adipose tissue contributes largely to the obesity-associated inflammation and is associated with low bone strength, small bone size, and increased hip fracture risk^22–24^—strengthening the notion that adipose tissue can affect bone negatively.

As many of the studies investigating the impact of obesity on bone are association studies, we aimed to experimentally test whether acute loss of adipose tissue, achieved through selective adipocyte ablation, would affect bone mass in mice. To acutely ablate adipocytes, we used transgenic FAT-ATTAC mice in which adipocyte-specific apoptosis can be induced through targeted activation of caspase 8^25^. We chose age-related obesity as model in this study primarily because of its resemblance with common human obesity^26^. Apart from analysis of bone mass, biomechanical properties of bone, bone turnover, and levels of peripheral (total and visceral adipose tissue) and bone marrow adipose tissue, we have also analyzed immune cells and inflammation as these factors can affect bone and are altered by adiposity and adipocyte apoptosis^25,27,28^.

## Methods

### Animals

The animal experiments were approved by the Gothenburg animal experimental ethics board (permit number: 194-15). The mice were housed under standard conditions of light and temperature at the animal facility at the Laboratory for Experimental Biomedicine, University of Gothenburg, Gothenburg, Sweden. Food (RM1; SDS Diets, Essex, UK) and water were provided ad libitum. To study the effect of acute adipose tissue loss, transgenic FAT-ATTAC mice were used^25^. In this inducible model, adipocyte numbers are reduced by an injection of dimerizer AP20187 (hereafter called dimerizer) that results in adipocyte (aP2-expressing cells)-specific apoptosis through targeted activation of caspase 8^25^. The present study followed relevant guidelines and regulations and it was carried out in compliance with the ARRIVE guidelines.

Dimerizer (ARIAD Pharmaceuticals, USA; 1 μg/g body weight) or vehicle (2% ethanol, 10% PEG-400, 2% Tween-20 in water) was injected intraperitoneally to male FAT-ATTAC mice at 5.5 or 9 months of age to allow for age-related obesity to occur before adipocyte apoptosis. The mice were randomized by weight to produce groups with equal mean body weight. The younger mice were used in a short-term experiment, terminated two weeks after induction of adipocyte apoptosis, while the older mice were used in a long-term experiment, terminated six weeks after induction of adipocyte apoptosis. At the time of termination, the mice were anesthetized with intraperitoneal injection with ketamine and dexmedetomidine before cervical dislocation. Quadriceps muscle and gonadal and retroperitoneal adipose tissue was dissected and weighed. To test whether the dimerizer had any off-target effects, we compared wild-type mice at 5.5 months of age that received vehicle or dimerizer. We could not find any differences between the mice regarding body composition, bone phenotype, or immune cell frequencies; confirming the specificity of the dimerizer (Supplementary Table S1).

### Dual-energy X-ray absorptiometry (DXA)

Total body areal bone mineral density (aBMD) and fat and lean mass were determined by a Lunar PIXImus mouse densitometer (Wipro GE Healthcare, Madison, WI, US) at 0, 1, and 2 weeks after adipocyte ablation in the short-term experiment and at 0, 2, 4, and 6 weeks after adipocyte ablation in the long-term experiment. Sedation was achieved by isoflurane inhalation, except at termination when an intraperitoneal injection of ketamine and dexmedetomidine was used.

### Gene expression analysis

For mRNA expression of tibial bone marrow, both ends of tibiae were cut and the bone marrow was spun out into a tube that was snap frozen and then stored in − 80 °C. Total RNA was extracted using TRIzol Reagent (Thermo Fisher Scientific, Waltham, MA, USA), RNeasy Mini Kit (Qiagen, Hilden, Germany), and RNase-Free DNase Set (Qiagen). cDNA synthesis was performed using the High-Capacity cDNA Reverse Transcription Kit (Thermo Fisher Scientific). Quantification of mRNA expression was performed by real time reverse transcriptase (RT)-PCR on a StepOnePlus Real-Time PCR System (Thermo Fisher Scientific) using the following pre-designed TaqMan Real-Time PCR Assays (Thermo Fisher Scientific): *Actb* (Mm00607939_s1) and *Adipoq* (Mm00456425_m1). The cDNA was run in duplicates along with the following controls: no RT control and non-template control. Gene expression values were calculated based on the ΔΔCt method^29^ so that the expression of the control group, the vehicle-treated mice, was set to 1 and the expression of the dimerizer-treated mice is relative to the control group and shown as fold change values.

### Computed tomography (CT)

Dissected femora, tibiae, and vertebrae L_5_ were placed in 10% (v/v) phosphate-buffered formalin for 2 days and then stored in 70% (v/v) ethanol. In tibiae and femora, the trabecular and cortical bone was analyzed using peripheral quantitative computed tomography (pQCT), as previously described^30^. In the tibia, the pQCT scans were positioned at a distance distal from the proximal growth plate corresponding to 2.6% of the total tibia length for trabecular bone scans and 30% for cortical bone scans. In the femur, the scans were positioned at a distance proximal from the distal growth plate corresponding to 3.0% of the total femur length for trabecular bone scans and 36% for cortical bone scans. In vertebrae L_5_, the trabecular bone in the vertebral body was analyzed by high-resolution micro-computed tomography (μCT), as previously described^31^.

### Biomechanical properties

After dissection, vertebra L_3_ and humeri were stored at − 20 °C. Just before biomechanical testing, the bones were thawed and remaining soft tissue was removed. Biomechanical properties were assessed in L_3_ vertebrae by a compression test and in humeral shafts by a three-point bending test. The vertebral body of L_3_ was loaded axially with a 2 mm flat-tipped press head when stabilized with a 1.4-mm-thick holder through the vertebral foramen. In the three-point bending test (span length 5.5 mm), humerus was loaded from the anterior side at the lower end of deltoid tuberosity. Loading speed for both L_3_ vertebra and humerus were 0.155 mm/sec with a mechanical testing machine (Instron 3366; Instron, Noorwood, MA, USA). Based on the computer-recorded load deformation raw data curves, produced by Bluehill 2 software version 2.6 (Instron), the results were calculated with MS-Excel macros.

### Serum analyses

Enzyme immunoassays were used to determine serum levels of the bone resorption marker C-terminal telopeptides of type I collagen (CTX-I) (RatLaps EIA, Immunodiagnostic Systems Nordic a/s, Copenhagen, Denmark), the bone formation marker Procollagen I Intact N-Terminal (PINP) (Rat/mouse PINP, Immunodiagnostic Systems), and the pro-inflammatory cytokine tumor necrosis factor (TNF alpha Mouse ProQuantum Immunoassay Kit, Invitrogen, Carlsbad, CA, USA) according to the manufacturer’s instructions.

### Flow cytometry analysis

Bone marrow cells from femur and splenocytes were isolated as previously described^32^. Cell surface markers were labeled with anti-CD19 PE (Becton Dickinson, BD, Franklin Lakes, NJ, USA), anti-CD3 APC Cy7 (BioLegend, San Diego, CA, USA), anti-CD4 V500 (BD), anti-CD8 PE Cy7 (eBioscience from Thermo Fisher Scientific, Waltham, MA, USA), anti-CD11b v500 (BD), anti-Gr-1 PerCP (BioLegend), anti-F4/80 APC Cy7 (BioLegend), anti-MCSF-R APC (BioLegend), anti-IgM PE (SouthernBiotech, Birmingham, AL, USA), anti-B220 PE Cy5 (BioLegend), mouse hematopoietic lineage antibody cocktail eFlour 450 (eBioscience), anti-CD45 PE (eBioscience), anti-cKit APC (BioLegend), and anti-Sca1 PE Cy7 (BioLegend). Singlet cells were gated using forward scatter height versus area. Lymphocytes were gated on singlet cells (based on forward and side scatter) and then T cells were defined as CD3^+^ lymphocytes, CD4^+^ T cells as CD4^+^CD3^+^ lymphocytes, CD8^+^ T cells as CD8^+^CD3^+^ lymphocytes, and B cells as CD19^+^ lymphocytes. B cells were further characterized in a separate panel where pro/pre B cells were defined as B220^+^IgM^+^ lymphocytes, immature B cells as B220^int^IgM^+^ lymphocytes, and recirculating B cells as B220^hi^IgM^+^ lymphocytes. Innate cells were gated on live singlet cells (based on forward and side scatter), and then granulocytes were defined as CD11b^+^F4/80^-^Gr-1^hi^, monocytes as CD11b^+^F4/80^+^Gr-1^int^, macrophages as CD11b^+^F4/80^+^Gr-1^−^, and pre-osteoclasts as CD11b^+^F4/80^+^Gr-1^int^MCSF-R^+^ cells. Hematopoietic cells were first gated on live singlet cells (based on forward and side scatter) and then defined as CD45^+^ cells while the hematopoietic stem cells were defined as CD45^+^Lin^-^c-Kit^+^Sca1^+^ cells. Samples were run on a BD FACS Verse and data was analyzed using the Flow Jo 10.2 software (3 Star Inc, Ashland, USA).

### Statistical analysis

Statistical analyses were performed using the SPSS software (version 25.0.0.0 for Windows). For comparisons of numeric data between two groups, the independent Student’s t-test was used unless Levene’s test revealed unequal variance, then the independent Welch’s t-test was used. Logarithmic transformations were used when appropriate to ensure normal distribution of data. All tests were two-sided. As the sample size was relatively small, we have verified that similar results are obtained by using the non-parametric Mann–Whitney test. Data are presented as scatter plots of individual data and a bar indicating the arithmetic mean or as the arithmetic mean ± SD unless otherwise stated. *P* < 0.05 was considered significant.

## Results

### Peripheral fat mass was quickly reduced following induction of adipocyte apoptosis

**Figure 1:**
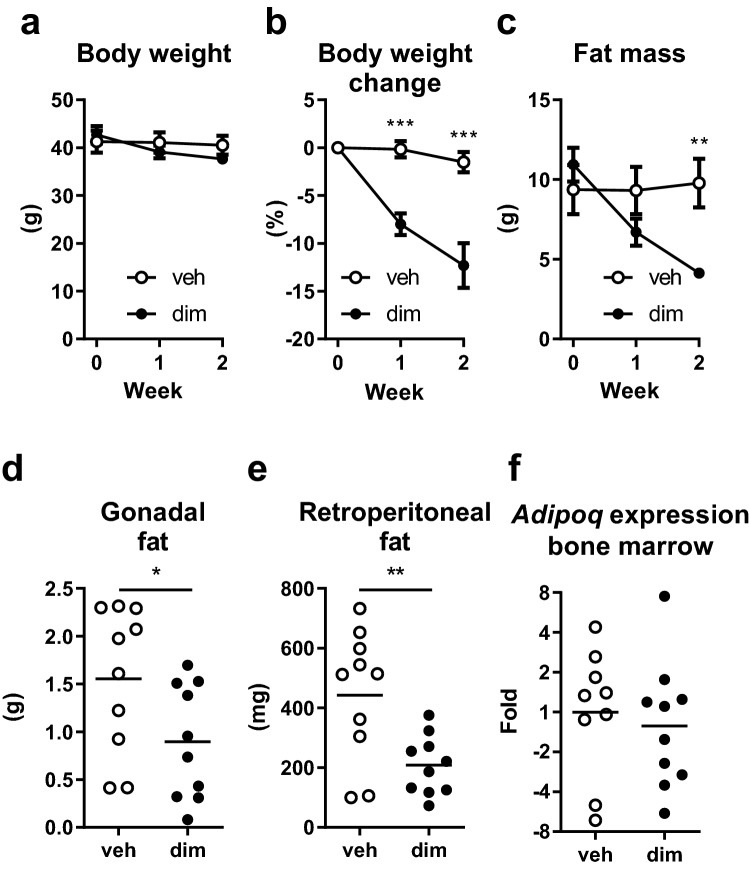
Peripheral fat mass was reduced two weeks after induction of adipocyte apoptosis. (**a**) Body weight, (**b**) percent body weight change, and (**c**) total fat mass, measured by DXA, at 0, 1, and 2 weeks after dimerizer injection. Weight of dissected (**d**) gonadal and (**e**) retroperitoneal fat pads 2 weeks after dimerizer injection. (**f**) Tibial bone marrow adiposity, assessed by gene expression of the adiponectin gene (*Adipoq*) 2 weeks after dimerizer injection. (**a**–**c**) Data are arithmetic mean ± sem. (**d**–**e**) Symbols in the scatter plots represent individual mice and the bars indicate arithmetic mean. (**f**) Symbols in the scatter plot represent individual mice and the bars indicate geometric mean. dim, dimerizer; veh, vehicle. *n* = 10 veh and 10 dim mice, except for (**f**) were n = 9 veh and 10 dim mice, due to laboratory error. (**a**–**b**, **d**, **f**) Student’s t-test. (**c**, **e**) Welch’s t-test. * *P* < 0.05, ** *P* < 0.01, and *** *P* < 0.001.

To determine whether acute loss of adipose tissue affects bone mass in mice with age-related obesity, adipocyte apoptosis was induced in FAT-ATTAC mice by an intraperitoneal dimerizer injection at 5.5 months of age. At this age, the mice had a mean body weight of 42 g (± 5.7 SD). The mice were followed for 2 weeks after induction of adipocyte apoptosis. At the end of this short-term experiment, dimerizer-treated mice lost 12.3% of their body weight (*P* = 0.002), while no significant weight loss was observed in vehicle-treated mice (Fig. 1a–b). As expected, the body weight loss in the dimerizer-treated mice was accompanied by a loss of total fat mass measured by DXA (Fig. 1c). The change in total fat mass, compared with baseline, was greater in dimerizer-treated mice than vehicle-treated mice at week 1 (veh: − 0.06 ± 0.95 g, n = 10, dim: − 4.2 ± 1.4 g, n = 10, *P* < 0.001, Student’s t-test) and at week 2 (veh: 0.04 ± 1.07 g, n = 10, dim: − 6.8 ± 2.4 g, n = 10, *P* < 0.001, Student’s t-test). Also, the dissected gonadal fat pads weighed 42% less and retroperitoneal fat pads weighed 53% less in the dimerizer-treated mice compared with the vehicle-treated mice (Fig. 1d–e). However, there were no difference between groups regarding bone marrow adiposity, as determined by gene expression analysis of *Adipoq* (encoding adiponectin) (Fig. 1f). There was also no difference in weight of the quadriceps muscle (veh: 247 ± 22 mg, n = 10, dim: 243 ± 23 mg, n = 10, *P* = 0.7, Student’s t-test).

### Short-term adipose tissue loss did not affect structural bone parameters

**Figure 2:**
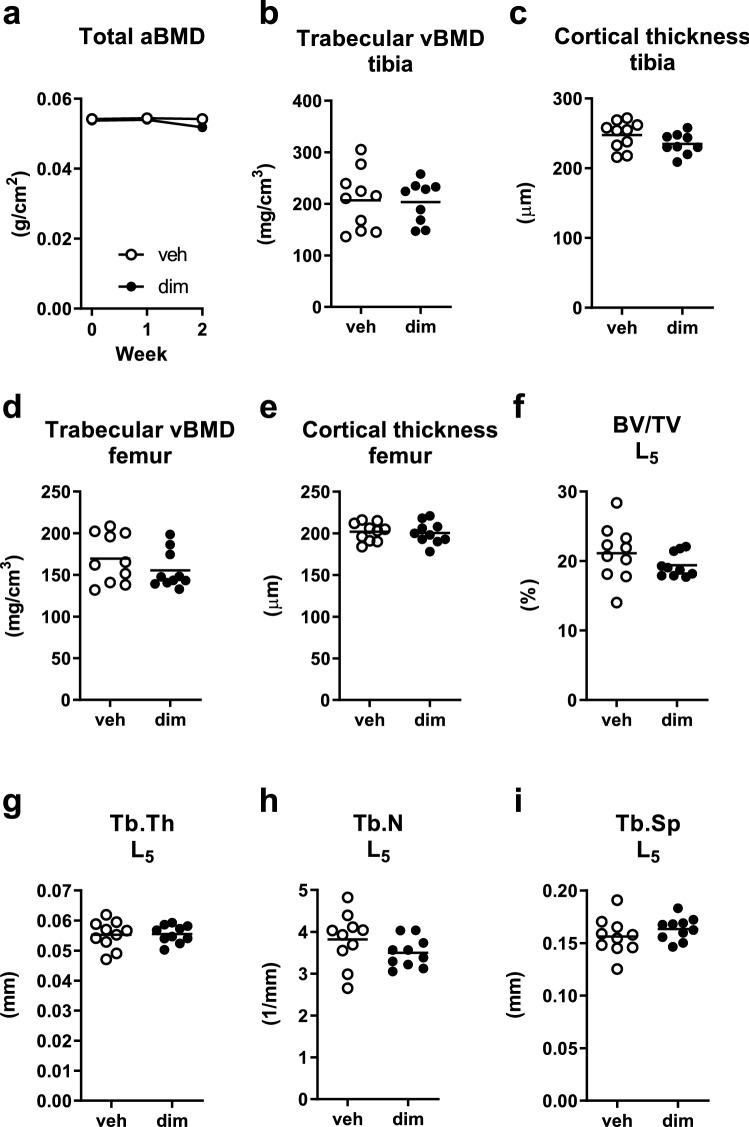
Short-term adipose tissue loss did not affect structural bone parameters. (**a**) Total areal bone mineral density (aBMD) measured by DXA at 0, 1, and 2 weeks after dimerizer injection. (**b**) Trabecular volumetric BMD (vBMD) of tibia, (**c**) cortical thickness of tibia, (d) trabecular vBMD of femur, and (**e**) cortical thickness of femur measured by pQCT 2 weeks after dimerizer injection. (**f**) Trabecular bone volume fraction (BV/TV), (**g**) trabecular thickness (Tb.Th), (**h**) trabecular number (Tb.N), (**i**) trabecular separation (Tb.Sp) of vertebra L_5_ measured by μCT 2 weeks after dimerizer injection. (**a**) Data are arithmetic mean ± sem. (**b**–**i**) Symbols in the scatter plots represent individual mice and the bars indicate arithmetic mean. dim, dimerizer; veh, vehicle. *n* = 10 veh and 10 dim mice (except for b where *n* = 9 dim mice due to laboratory error). (**a**–**i**) Student’s t-test.

To follow the impact of acute adipose tissue loss on bone throughout the experiment, we measured total areal bone mineral density (aBMD) by DXA at baseline and 1 and 2 weeks post dimerizer/vehicle administration. We found no effect of adipose tissue loss on total aBMD at any time point (Fig. 2a), although there was a trend towards decreased total aBMD at the last time point (*P* = 0.053). The change in total aBMD, compared with baseline, did not differ at week 1 (veh: 0.2 ± 1.7 g/cm^3^, n = 10, dim: 0.2 ± 1.3 g/cm^3^, n = 10, *P* = 0.99, Student’s t-test) but was greater in dimerizer-treated mice than vehicle-treated mice at week 2 (veh: 0.01 ± 1.9 g/cm^3^, n = 10, dim: − 2.0 ± 2.0 g/cm^3^, n = 10, *P* = 0.04, Student’s t-test). More careful bone phenotyping using pQCT showed that there were no differences between groups in the trabecular volumetric BMD (vBMD) or in the cortical thickness in the tibia and femur (Fig. 2b–e). Furthermore, μCT analysis of vertebra L_5_ showed that there were no differences between groups in trabecular bone volume, trabecular thickness, trabecular number, or trabecular separation (Fig. 2f–i).

### Short-term adipose tissue loss resulted in altered frequencies of lymphocyte populations

As there is an interplay between immune cells, adipocytes, and bone^27,28^, we investigated the frequency of immune cells within the bone marrow and spleen. In the bone marrow, short-term adipose tissue loss did not affect the cellularity or the frequency of hematopoietic cells, hematopoietic stromal cells, or lymphocytes (Table 1). However, the frequencies of lymphocyte subpopulations were skewed with a decreased frequency of T cells in dimerizer-treated mice, and an increased frequency of B cells (Table 1). For the T cells, there was a general decrease of different subpopulations as the frequencies of CD4^+^ T cells, CD8^+^ T cells, and CD4^-^CD8^-^ T cells were decreased in dimerizer-treated mice compared with vehicle-treated controls (Table 1). For the B cells, there was mainly an increase in the frequency of pro/pre B cells, while there was no difference in immature B cells or recirculating B cells. Although there were differences in the frequencies of some cell populations within the acquired immune system, there were no differences in the frequency of innate immune populations—monocytes, macrophages, and granulocytes—or pre-osteoclasts (Table 1).

**Table 1:** Total bone marrow cellularity and frequencies of cell populations within the bone marrow.

Cell population	tg veh	tg dim	*P*-value
Total cellularity (× 10^6^ cells)	12.7 ± 3.3	13.6 ± 2.2	0.512
Lymphocytes (% of alive)	41.0 ± 5.7	45.8 ± 5.0	0.107
T cells (% of lymphocytes)	10.6 ± 1.7	7.9 ± 1.0	0.002
CD4^+^ T cells (% of lymphocytes)	1.4 ± 0.5	1.0 ± 0.2	0.018
CD8^+^ T cells (% of lymphocytes)	2.5 ± 0.6	1.8 ± 0.5	0.031
CD4^-^CD8^-^ T cells (% of lymphocytes)	6.6 ± 1.1	5.1 ± 0.7	0.006
B cells (% of lymphocytes)	30.5 ± 8.1	41.0 ± 6.0	0.013
Pro/pre B cells (% of lymphocytes)	20.8 ± 3.6	33.7 ± 4.8	< 0.001
Immature B cells (% of lymphocytes)	2.5 ± 0.8	3.3 ± 0.6	0.058
Recirculating B cells (% of lymphocytes)	7.6 ± 3.0	7.3 ± 4.2	0.876
Hematopoietic cells (% of alive)	80.4 ± 10.1	85.8 ± 5.0	0.153
Hematopoietic stem cells (% of alive)	0.33 ± 0.10	0.34 ± 0.06	0.752
Neutrophils (% of alive)	21.6 ± 9.8	22.3 ± 8.0	0.872
Monocytes (% of alive)	1.7 ± 0.4	1.6 ± 0.5	0.662
Macrophages (% of alive)	1.0 ± 0.2	1.1 ± 0.4	0.835
Pre-osteoclasts (% of alive)	0.16 ± 0.07	0.15 ± 0.05	0.834

Data are arithmetic mean ± standard deviation. tg, transgene; veh, vehicle; dim, dimerizer n = 10 veh and 10 dim mice. Student’s t-test.

In the spleen, there was no difference in cellularity between groups, but compared to vehicle-treated mice, dimerizer-treated mice had a lower frequency of lymphocytes, attributed to the decreased frequency of T cells and unaltered frequency of B cells (Table 2). For the T cells, there was a decrease in the frequency of CD4^+^ and CD8^+^ T cells in dimerizer-treated compared with vehicle-treated mice, but there was no difference in the frequency of the CD4^-^CD8^-^ T cell population (Table 2).

**Table 2:** Total spleen cellularity and frequencies of cell populations within the spleen.

Cell population	tg veh	tg dim	*P*-value
Total cellularity (× 10^6^ cells)	29.8 ± 14.5	34.9 ± 14.4	0.443
Lymphocytes (% of alive)	56.8 ± 4.6	51.1 ± 6.7	0.042
T cells (% of lymphocytes)	35.9 ± 4.7	29.8 ± 3.2	0.003
CD4^+^ T cells (% of lymphocytes)	19.2 ± 3.1	15.0 ± 1.6	0.001
CD8^+^ T cells (% of lymphocytes)	11.2 ± 1.8	8.7 ± 1.3	0.002
CD4^-^CD8^-^ T cells (% of lymphocytes)	4.8 ± 1.1	5.1 ± 1.2	0.585
B cells (% of lymphocytes)	44.9 ± 3.9	45.0 ± 3.9	0.937

Data are arithmetic mean ± standard deviation. tg, transgene; veh, vehicle; dim, dimerizer n = 10 veh and 10 dim mice. Student’s t-test.

### Adipocyte apoptosis resulted in long-term loss of peripheral adipose tissue

**Figure 3:**
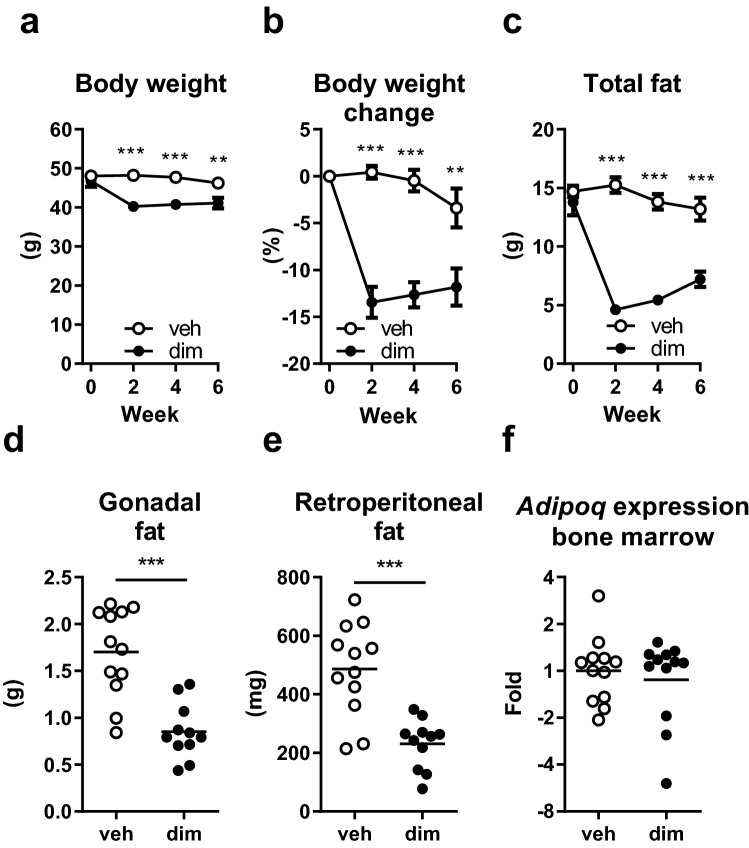
Adipocyte apoptosis resulted in long-term loss of peripheral adipose tissue. (**a**) Body weight, (**b**) percent body weight change, and (**c**) total fat mass, measured by DXA, at 0, 2, 4, and 6 weeks after dimerizer injection. Weight of dissected (**d**) gonadal and (**e**) retroperitoneal fat pads 6 weeks after dimerizer injection. (**f**) Tibial bone marrow adiposity, assessed by gene expression of the adiponectin gene (*Adipoq*) 6 weeks after dimerizer injection. (**a**–**c**) Data are arithmetic mean ± sem. (**d**–**e**) Symbols in the scatter plots represent individual mice and the bars indicate arithmetic mean. (**f**) Symbols in the scatter plot represent individual mice and the bars indicate geometric mean. dim, dimerizer; veh, vehicle. *n* = 12 veh and 12 dim mice. (**a**–**e**) Student’s t-test. *** *P* < 0.001.

To determine the long-term effects of adipocyte apoptosis on bone mass, the mice (body weight 47 g ± 4.1 SD) were followed for six weeks following dimerizer injection. Similar to the short-term study, body weight was lost to a larger extent in dimerizer-treated mice compared with vehicle-treated mice, with the largest loss observed after two weeks (Fig. 3a). At this time point, the dimerizer-treated mice had lost 13% of their body weight, while the vehicle-treated mice maintained their body weight (Fig. 3b). Using DXA, total body fat mass quickly dropped in dimerizer-treated mice, with the lowest levels at two weeks when they had 68% lower total fat mass than vehicle-treated mice (Fig. 3c). The change in total fat mass, compared with baseline, was greater in dimerizer-treated mice than vehicle-treated mice at week 2 (veh: 0.5 ± 1.0 g, n = 12, dim: − 9.2 ± 3.0 g, n = 12, *P* < 0.001, Student’s t-test), week 4 (veh: − 0.9 ± 2.5 g, n = 12, dim: − 8.3 ± 3.3 g, n = 12, *P* < 0.001, Student’s t-test), and week 6 (veh: − 1.5 ± 3.7 g, n = 12, dim: − 6.6 ± 3.5 g, n = 12, *P* = 0.002, Student’s t-test). Compared with vehicle-treated mice, the dissected gonadal fat pads weighed 48% less in dimerizer-treated mice, and the dissected retroperitoneal fat pads weighed 52% less in dimerizer-treated mice (Fig. 3d–e). However, there was no difference between groups regarding bone marrow adiposity, as determined by gene expression analysis of *Adipoq* (encoding adiponectin) (Fig. 3f). There was also no difference in weight of the quadriceps muscle (veh: 247 ± 29 mg, n = 12, dim: 264 ± 21 mg, n = 12, *P* < 0.11, Student’s t-test) or lean mass at termination (veh: 30.1 ± 2.1 g, n = 12, dim: 30.9 ± 2.6 g, n = 12, *P* < 0.37, Student’s t-test).

To assess the inflammatory status of the mice, we quantified serum levels of the pro-inflammatory cytokine tumor necrosis factor (TNF) at baseline and 6 weeks following induction of adipocyte apoptosis. The levels were generally low, with no differences at baseline (veh: 6.2 ± 6.0 pg/ml, n = 12, dim: 4.2 ± 2.4 pg/ml, n = 12, *P* = 0.3, Student’s t-test) or at 6 weeks (veh: 4.9 ± 6.4 pg/ml, n = 12, dim: 4.3 ± 2.7 pg/ml, n = 12, *P* = 0.7, Student’s t-test), nor was there a difference in the change in TNF during the experiment (veh: − 1.2 ± 9.5 pg/ml, n = 12, dim: 0.1 ± 3.3 pg/ml, n = 12, *P* = 0.6, Student’s t-test).

### Long-term adipose tissue loss did not affect bone

**Figure 4:**
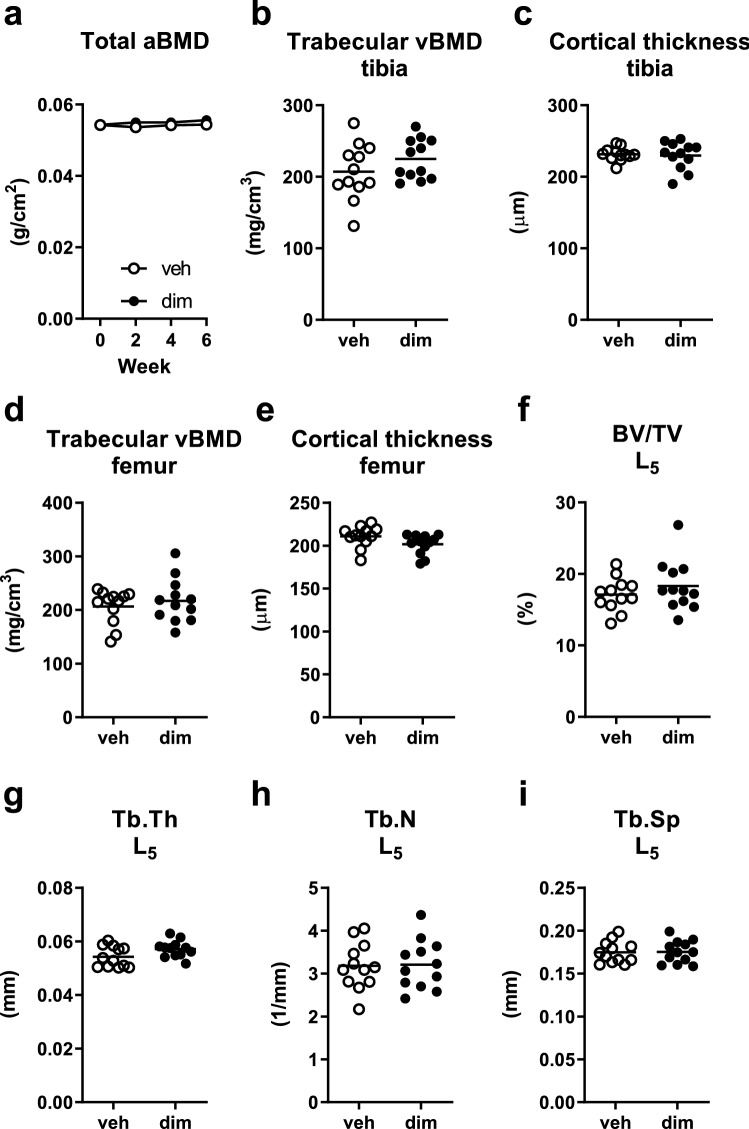
Long-term adipose tissue loss did not affect structural bone parameters. (**a**) Total areal bone mineral density (aBMD) measured by DXA at 0, 1, and 2 weeks after dimerizer injection. (**b**) Trabecular volumetric BMD (vBMD) of tibia, (**c**) cortical thickness of tibia, (**d**) trabecular vBMD of femur, and (**e**) cortical thickness of femur measured by pQCT 6 weeks after dimerizer injection. (**f**) Trabecular bone volume fraction (BV/TV), (g) trabecular thickness (Tb.Th), (**h**) trabecular number (Tb.N), (**i**) trabecular separation (Tb.Sp) of vertebra L_5_ measured by μCT 6 weeks after dimerizer injection. (**a**) Data are arithmetic mean ± sem. (**b–i**) Symbols in the scatter plots represent individual mice and the bars indicate arithmetic mean. dim, dimerizer; veh, vehicle. *n* = 12 veh and 12 dim mice (except for b where *n* = 11 dim mice due to laboratory error). (**a**–**i**) Student’s t-test.

Despite allowing a longer time for bone formation in the long-term experiment, there was no difference between groups in total aBMD at any time point (Fig. 4a). The change in total aBMD, compared with baseline, did not differ between groups at week 2 (veh: − 0.6 ± 2.4 g/cm^3^, n = 12, dim: 0.7 ± 2.4 g/cm^3^, n = 12, *P* = 0.2, Student’s t-test), week 4 (veh: − 0.06 ± 1.8 g/cm^3^, n = 12, dim: 0.7 ± 2.1 g/cm^3^, n = 12, *P* = 0.4, Student’s t-test), or week 6 (veh: 0.09 ± 2.1 g/cm^3^, n = 12, dim: 1.3 ± 2.2 g/cm^3^, n = 12, *P* = 0.2, Student’s t-test). Even when more careful phenotyping, using pQCT, of tibia and femur was employed, trabecular vBMD and cortical thickness did not differ between groups (Fig. 4b–e). Similar to the other bone measurements, , μCT analysis of vertebra L_5_ could not detect any differences in trabecular bone parameters between groups (Fig. 4f–i).

**Figure 5:**
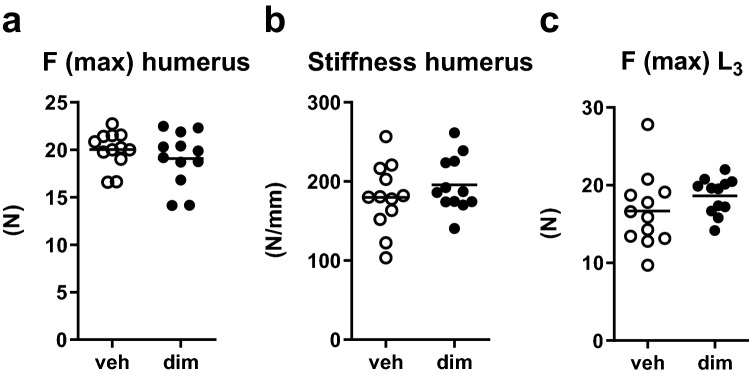
Long-term adipose tissue loss did not affect biomechanical properties of bone. (**a**) Ultimate strength (F (max)) and (**b**) stiffness of the humerus, determined by 3-point bending, and (**c**) ultimate strength of vertebra L_3_, determined by compression test. Symbols in the scatter plots represent individual mice and the bars indicate arithmetic mean. dim, dimerizer; veh, vehicle. *n* = 12 veh and 12 dim mice. (**a**–**c**) Student’s t-test.

To exclude the possibility that the bone of dimerizer-treated mice differed from vehicle-treated mice with regards to parameters other than the structural parameters, we tested the biomechanical properties. The biomechanical properties of cortical bone, assessed by the maximal load (ultimate strength) and stiffness of humerii, did not differ between groups (Fig. 5a–b). Likewise, the biomechanical properties of trabecular bone, assessed by the maximal load in vertebra L_3_, did not differ between groups (Fig. 5c).

Furthermore, we also assessed bone turnover by analyzing the serum levels of the bone formation marker PINP and the bone resorption marker CTX-1. These analyses show that there were no differences between groups regarding bone formation and resorption at baseline (0 weeks) or after 6 weeks, nor was there a difference in the change in these markers during the experiment (Table 3).

**Table 3:** Serum levels of bone turnover markers.

Bone turnover marker	tg veh	tg dim	*P* value
PINP (ng/ml)			
0 weeks	25.6 ± 6.7	27.7 ± 6.3	0.429
6 weeks	27.3 ± 14.0	23.0 ± 4.5	0.321
Change, compared with 0 weeks	1.6 ± 16.9	-4.8 ± 7.3	0.239
CTX-1 (ng/ml)			
0 weeks	14.5 (11.5–18.2)	12.9 (10.9–15.3)	0.379
6 weeks	11.1 (8.5–14.5)	9.0 (7.3–11.1)	0.179
Change, compared with 0 weeks	−3.3 ± 9.1	−3.9 ± 4.4	0.833

Data are arithmetic mean ± standard deviation, except for CTX-1 at 0 and 6 weeks that are geometric mean (95% confidence interval). veh, vehicle; dim, dimerizer; PINP, Procollagen I Intact N-Terminal; CTX-1, C-terminal telopeptides of type I collagen. n = 12 veh and 12 dim mice. Student’s t-test.

## Discussion

In the present study, we sought to determine the effect of acute adipose tissue loss on bone mass in age-related obese mice. Although obesity has been considered to protect the bone, its protective role has been questioned and several studies have shown negative associations between adipose tissue and bone mass^23,33,34^. However, we did not detect any effect of the acute adipose tissue loss on bone mass, despite substantial loss of peripheral adipose tissue, and analyses of several different bone sites as well as both trabecular and cortical bone parameters. In line with these data, we did not find any differences in biomechanical properties of bone or markers of bone turnover. Thus, in our setting, adipose tissue loss does not affect bone.

As increased adiposity is associated with low bone mass in humans^23,24,33–35^, proposedly due to the obesity-induced inflammation^17,21^, we were surprised by the lack of bone phenotype in our mice following adipose tissue loss. However, as we observed low levels of serum TNF at baseline, and no additional reduction following adipose tissue loss, the possible benefit of the adipose tissue loss may be too low to increase the bone mass, especially when these benefits may have been counteracted by the negative impact of decreased mechanical loading caused by the weight loss and by a loss of the bone-protective hormone estrogen; in part derived from adipose tissue^2,36^. As serum estrogen levels in male mice are very low, especially in older mice where they are virtually unmeasurable, we did not attempt to measure estrogens in this project^37^.

In humans, the closest to induction of acute adipose depletion is bariatric surgery. During this procedure, the body weight and adipose mass drop rapidly^38^. Despite the negative associations between adipose tissue and bone mass, neither adipose tissue loss in our study nor following bariatric surgery is associated with improved bone mass; in fact, bariatric surgery is associated with bone loss^39^. The different results may be due to the relationship between the negative bone effects caused by decreased mechanical loading and the beneficial bone effects from decreased obesity-associated inflammation. Furthermore, bariatric surgery is not merely an ablation of adipose tissue and this procedure, therefore, differs substantially from our experimental setting where adipose tissue is lost due to specific induction of apoptosis in adipocytes in contrast to rewiring of the gut. Following bariatric surgery, nutrient uptake, feeding behavior, and gut hormone levels are altered^40,41^; alterations that may impact bone health.

The relationship between adipose tissue and bone has previously been assessed in experimental studies using mouse models that lack adipose tissue to various degrees. In line with our data, mice with germline knockout of caveolin-1 had decreased visceral adipose tissue mass and no bone phenotype^42^ despite metabolic dysfunction^43^. However, another mouse model lacking adipose tissue due to germline knockout of *Ptrf* had a mild bone phenotype in males where only the cortical mineral content of the tibia was increased, while the females had a more pronounced bone phenotype where also the trabecular bone volume fraction and the trabecular number were increased^42^. Similarly, fat-free and severely insulin resistant A-ZIP/F-1 transgenic mice had an increased bone mass^44^. Furthermore, fat-free mice generated by expression of diphtheria toxin in adipocytes had a large improvement of their bone, with approximately 300% increase in the trabecular bone volume fraction^45^.

All the above-mentioned mouse models with adipose tissue loss have in common that adipose tissue is absent or partly absent throughout the life of the mice. To avoid any developmental effects of adipose tissue loss, we used the inducible FAT-ATTAC mouse model for adipose tissue ablation in adulthood^25^. A similar approach was recently used by Zou et al. when they ablated adipocytes by administering diphtheria toxin to adult mice expressing the diphtheria toxin receptor in adipocytes (DT/DTR^ADQ^ model)^46^. Similar to their germline adipocyte depletion^45^, but in contrast to our findings, adipocyte ablation induced by diphtheria toxin injections strongly induced bone formation (DT/DTR^ADQ^ model), while bone was unaffected when adipocytes were ablated following a tamoxifen injection that activated diphtheria toxin expression within adipocytes (TAM/DT^ADQ ER^ model)^46^. One suggested explanation for the difference between the two models of inducible adipocyte ablation in the paper by Zou et al. is the expression of diphtheria toxin receptor in adipocytes in the DT/DTR^ADQ^ model, the model with a bone phenotype. Diphtheria toxin receptor and its cleaved products are released upon adipocyte death, and they can function as growth factors by activating EGFR signaling. Indeed, bone formation was severely blunted following treatment with an EGFR inhibitor^46^, suggesting that the model of fat ablation itself is important to consider as it may result in effects of its own. An advantage with the AP20187 dimerizer that we used for adipocyte apoptosis is that we and others have found no unspecific effects, for example regarding bone and body composition^47^.

Although the main aim of our study was to test the effect of peripheral adipose tissue loss on bone, we also assessed whether bone marrow adiposity was decreased in our FAT-ATTAC mice. As the lack of bone marrow adipocytes has been suggested to explain the difference between some strains with and without a bone phenotype (Caveolin-1 vs- *Ptrf* knockouts and tamoxifen vs diphtheria toxin induction)^42,46^, a lack of bone phenotype in our FAT-ATTAC mice may be explained by the ineffective ablation of bone marrow adipocytes. However, a pilot study indicates that apoptosis is indeed induced in bone marrow adipocytes of FAT-ATTAC mice (two days after dimerizer injection), as the vast majority of the bone marrow adipocytes were devoid of perilipin surface stain, demonstrating that those adipocytes were dead (Grahnemo, unpublished data). These data may indicate that bone marrow adipocytes regenerate more quickly than the peripheral adipocytes in the gonadal and retroperitoneal adipose tissue. Furthermore, the findings by us and others suggest that changes in bone marrow adipose tissue are more important for bone than changes in peripheral adipose tissue.

A limitation of our study is that we assessed bone turnover using serum markers of bone formation and resorption instead of the gold-standard, histomorphometry. A second limitation is that we only used the mild age-induced obesity model and not the commonly used and more severe high-fat diet-induced obesity model, to allow easier comparisons with other studies. Another limitation is that we only followed the mice for two or six weeks. Although bone changes can occur in two weeks or less^48–50^, it is possible that our follow-up time was insufficient considering the slower bone formation in older animals. However, we did not extend the time further as the adipose tissue started to regenerate in the FAT-ATTAC mouse model, an inducible and reversible model of fat ablation^25^. As for all transgenic models, there is a matter of specificity of the promotor. In the FAT-ATTAC mice, adipocyte specificity is achieved by the Fabp4-promotor that induce high expression of the transgene in white adipose tissue and lower levels in brown adipose tissue, with resulting apoptosis^25^. Potentially, there is also some expression in macrophages—possibly also in bone resorbing osteoclasts as these two cell types share a common origin—although the levels may be too low to affect the function of the cells^51^. We find that an effect of the transgene in osteoblasts is very unlikely, as single-cell sequencing RNA data showed that endogenous Fabp4 is expressed at extremely low levels in osteoblasts^52^. Although the literature directly (adipocytes) and indirectly (bone cells) confirm specificity of the adipocyte apoptosis, it is a limitation that we have not confirmed specificity ourselves. Apart from adipocyte apoptosis, adipose tissue loss may also be induced by lipolysis and the subsequent reduction of adipocyte size. However, as adipocyte apoptosis and adipocyte lipolysis are two fundamentally different processes, conclusions regarding the effect of lipolysis-induced adipose tissue loss on bone cannot be based on this study.

Similar to caveolin-1 knockout mice, we managed to deplete gonadal tissue to approximately 50%, without a resulting bone phenotype^42^. All the other mentioned mouse models achieved a higher level of depletion, and all but the tamoxifen-inducible adipose depletion model had an improved bone phenotype. Potentially, a more severe adipose tissue depletion, which also includes the loss of bone marrow adipose tissue, could have increased the chance of a bone phenotype for our FAT-ATTAC mice, but may also be less physiologically relevant, resulting in severe metabolic side effects^53^. Alternatively, obesity induced by high-fat diet instead of age may have resulted in more severe obesity and obesity-induced inflammation, with for example increased levels of TNF, and thus subsequently a better bone-protective effect upon adipose tissue loss.

In conclusion, the impact of obesity and adipose tissue on bone is still incompletely understood. Several mouse models have tried to explore this issue, each with its pros and cons. In our study, inducible adipose tissue ablation in mice with age-related obesity did not affect bone. Future studies are warranted to test whether adipose tissue loss is beneficial in obesity with more pronounced metabolic and adipose tissue dysfunction.

## Supplementary Information

Supplementary Information.
