# Supplementary material for: Acute fat loss does not affect bone mass
Source: Sci Rep. 2021 Jul 8;11:14177. doi: 10.1038/s41598-021-93450-y (PMC8266894; doi:10.1038/s41598-021-93450-y)
Supplement: Supplementary file 1 — Supplementary Information. [file 41598_2021_93450_MOESM1_ESM.pdf]

Acute fat loss does not affect bone mass

Marie K Lagerquist, Karin L Gustafsson, Petra Henning, Helen Farman, Jianyao Wu, Klara Sjögren, Antti Koskela, Juha Tuukkanen, Claes Ohlsson, Ingrid Wernstedt Asterholm[#], Louise Grahnemo[#]

[#]equal contribution

Supplemental Table S1

	wt veh		wt dim		
	**mean**	**SD**	**mean**	**SD**	**P-value**
**Body composition**					
Body weight, week 0 (g)	40.5	5.5	42.1	4.6	0.415
Body weight, week 1 (g)	40.3	5.0	42.0	4.5	0.383
Body weight, week 2(g)	39.7	4.2	40.9	4.1	0.490
Body weight change, week 1 (%)	-1.0	3.2	-1.7	2.4	0.503
Body weight change, week 2 (%)	-1.5	4.8	-2.9	3.8	0.401
Fat mass, week 0 (g)	9.5	3.7	10.5	3.7	0.499
Fat mass, week 1 (g)	9.1	3.2	9.5	3.0	0.710
Fat mass, week 2 (g)	9.6	2.9	10.2	3.2	0.609
Gonadal fat (g)	1.7	0.5	1.7	0.5	0.886
Retroperitoneal fat (g)	0.44	0.14	0.45	0.13	0.921
**Bone variables**					
Total aBMD, week 0 (mg/cm^2)	549.2	2.5	55.0	2.2	0.911
Total aBMD, week 1 (mg/cm^2)	54.3	3.5	55.2	2.5	0.431
Total aBMD, week 2 (mg/cm^2)	53.9	3.3	55.2	2.1	0.273
Trabecular vBMD tibia (mg/cm^2)	286.3	48.5	286.0	46.5	0.987
Cortical thickness tibia (mm)	231.7	17.1	234.0	18.2	0.742
Trabecular vBMD femur (mg/cm^3)	287.9	67.9	311.7	76.2	0.398
Cortical thickness femur (mm)	207.1	15.7	209.7	13.8	0.664
BV/TV L_5 (%)	23.8	3.8	25.1	2.7	0.340
Trabecular thickness L_5 (mm)	50.8	3.6	51.0	3.8	0.862
Trabecular number L_5 (1/mm)	4.7	0.7	4.9	0.4	0.310
Trabecular separation L_5 (mm)	149.9	16.3	145.9	12.3	0.483
**Bone marrow cells**					
Total cellularity ($\times 10^6$ cells)	15.3	4.3	14.2	3.8	0.486
Lymphocytes (% of alive)	47.0	6.6	42.8	9.1	0.213
T cells (% of lymphocytes)a	14.6	3.5	14.6	5.5	0.970
$CD4^+$ T cells (% of lymphocytes)[a]	3.1	1.3	2.7	1.4	0.512
$CD8^+$ T cells (% of lymphocytes)[a]	3.7	1.2	4.1	1.4	0.430
$CD4^-CD8^-$ T cells (% of lymphocytes)[a]	7.8	1.4	7.8	3.2	0.965
B cells (% of lymphocytes)[a]	31.2	6.2	30.2	7.9	0.737
Pro/pre B cells (% of lymphocytes)[b]	17.8	4.4	18.1	3.1	0.824
Immature B cells (% of lymphocytes)[b]	2.2	0.6	2.3	0.5	0.506
Recirculating B cells (% of lymphocytes)[b]	15.1	3.2	14.2	3.2	0.513
Hematopoietic cells (% of alive)	83.8	6.5	81.8	10.0	0.539
Hematopoietic stromal cells (% of alive)	0.35	0.15	0.38	0.23	0.763
Neutrophils (% of alive)	24.3	5.8	26.4	10.6	0.516
Monocytes (% of alive)	1.8	0.4	1.8	0.5	0.923
Macrophages (% of alive)	1.2	0.5	1.3	1.0	0.829
Pre-osteoclasts (% of alive)	0.25	0.19	0.28	0.33	0.808
**Spleen cells**					
Lymphocytes (% of alive)	55.2	8.6	57.5	7.8	0.466
T cells (% of lymphocytes)	39.5	5.7	39.9	5.7	0.852
$CD4^+$ T cells (% of lymphocytes)	20.7	3.6	20.8	3.9	0.949
$CD8^+$ T cells (% of lymphocytes)	12.6	2.9	13.1	3.9	0.733
$CD4^-CD8^-$ T cells (% of lymphocytes)	5.4	2.0	5.3	2.7	0.985
B cells (% of lymphocytes)	43.9	6.0	42.8	8.5	0.697

Data are arithmetic mean ± standard deviation (SD). wt, wild type; veh, vehicle; dim, dimerizer; BMD, bone mineral density; aBMD, areal BMD; vBMD, volumetric BMD; BV/TV, bone mineral per tissue volume. n = 15 veh and 12 dim mice, unless otherwise specified. Student's t-test. [a]n=13 veh and 10 dim mice. [b]n=14 veh and 11 dim mice. Missing values are due to laboratory error.
